# Antibiotic resistance profile in sediments and bacterial isolates from a human-impacted mangrove from Bahía Magdalena, Mexico

**DOI:** 10.1093/sumbio/qvae010

**Published:** 2024-04-29

**Authors:** Stephany García-Martínez, Karen A Zapién-Chavarría, Blanca E Rivera-Chavira, Jaime R Adame-Gallegos, Oskar A Palacios, Guadalupe V Nevárez-Moorillón

**Affiliations:** Facultad de Ciencias Químicas, Universidad Autónoma de Chihuahua. Circuito Universitario s/n, Campus II. zip code 31125 Chihuahua, Chihuahua 31125, México; Facultad de Ciencias Químicas, Universidad Autónoma de Chihuahua. Circuito Universitario s/n, Campus II. zip code 31125 Chihuahua, Chihuahua 31125, México; Facultad de Ciencias Químicas, Universidad Autónoma de Chihuahua. Circuito Universitario s/n, Campus II. zip code 31125 Chihuahua, Chihuahua 31125, México; Facultad de Ciencias Químicas, Universidad Autónoma de Chihuahua. Circuito Universitario s/n, Campus II. zip code 31125 Chihuahua, Chihuahua 31125, México; Facultad de Ciencias Químicas, Universidad Autónoma de Chihuahua. Circuito Universitario s/n, Campus II. zip code 31125 Chihuahua, Chihuahua 31125, México; Facultad de Ciencias Químicas, Universidad Autónoma de Chihuahua. Circuito Universitario s/n, Campus II. zip code 31125 Chihuahua, Chihuahua 31125, México

**Keywords:** antibiotic-resistant bacteria, antibiotic resistance genes, mangroves, One Health, Baja Calfornia, Mexico

## Abstract

Antibiotic-resistant bacteria (ARB) and antibiotic-resistance genes (ARGs) in the environment are considered emerging contaminants. One of the ecosystems that can harbor resistant bacteria and genes is mangroves. This report aims to assess ARB and ARG's presence at two mangrove locations in Bahia Magdalena, Mexico. Bacterial isolates were selected based on their phenotypic profiles of antibiotic resistance. DNA from mangrove sediments and bacterial isolates was tested for *int*1 and antibiotic resistance genes by final point PCR. From 90 ARB isolates, the phenotypic profile showed resistance to ciprofloxacin (90%), cefotaxime (85.7%), and ampicillin (78.6%), while the beta-lactamase gene (*bla*CTX-M) was present in 72.9% of the isolates. Antibiotic resistance genes identified in sediments were found even at 30–50 cm depth, but the integron Class gen (*int*1) was found mainly in shallow samples. Principal component analysis showed a close relationship between the integron gene, vancomycin, and sulfonamides resistance genes. No correlation was found between the phenotypic and genotypic profiles of the bacterial isolates. Also, no differences were observed between the two mangrove ecosystems studied. The impact of anthropogenic activities was equally significant on both sites. The role of mangrove ecosystems in the dispersion of antibiotic resistance needs to be further explored.

**Sustainability Statement**

The global dispersion of antibiotic-resistant bacteria (ARB) a and antibiotic-resistance genes (ARGs) directly impacts Human Health but also concerns plants' and animals' well-being. Antibiotic resistance in clinical settings can directly relate to Sustainable Development Goal #3 (Good Health and Well-being) and Goal #1 (End of poverty). However, when the problem is considered from a systematic perspective, the problems related to the health of the environment and the animals are connected to Human Public Health. The One Health approach believes that human health is closely connected to the health of animals and the ecosystem, and among their main concerns is the dispersion of antibiotic resistance due to human-related activities. Therefore, Sustainable Goal #6 (Clean Water and Sanitation), Goal #14 (Life below water), and Goal # 15 (Life on land) will also be impacted. The present report describes the effects of human activities on the presence of antibiotic-resistant bacteria and antibiotic-resistance genes in a Mangrove site in Mexico.

## Introduction

Mangroves are salt-tolerant forests located in the transition between marine and terrestrial ecosystems. They are highly productive ecosystems with a wide diversity of salt-tolerant organisms; they have been reported to store an average of 1023 Mg of carbon per hectare (Donato et al. 2011). Mangroves are located in tropical areas, and based on the extension covered, Mexico is the fourth most-vast country with mangroves, with a predominance of the following mangrove species: *Rhizophora mangle, Laguncularia racemosa, Avicennia germinans*, and *Canocarpus erectus* (Rodríguez et al. 2018). The Gulf of California is considered one of the most productive and diverse areas in the Northwest of Mexico. It is a semiarid to an arid area with a subtropical climate but is also one of the regions with more economic growth, agriculture, and aquaculture as primary activities. Tourism and urban development are also rising (López-López 2013).

On the other hand, as in many other ecosystems, anthropogenic activities have highly affected mangroves (Imchen et al. 2019), including releasing industrial contaminants, fertilizers, and pesticides. Discharging wastewater in the mangrove ecosystem increases the organic matter content and allows the growth of microorganisms such as human pathogens and fecal coliforms, which would not be easily found otherwise (Cabral et al. 2011). Among the organic contaminants that can reach mangroves are antibiotic residues from urban settings or agricultural activities. Therefore, the presence of antibiotic-resistant bacteria (ARB) and antibiotic-resistance genes (ARGs) is not only related to health-related consumption of antibiotics but to other anthropogenic activities. ARB are not only pathogenic organisms, but the resistance to antibiotics can be transferred to microbial species found in the environment, which are, in turn, reservoirs of ARG (Che et al. 2019, Imchen et al. 2019).

The determination of ARB and ARG has been proposed as an alternative urbanization indicator since antibiotic resistance is related to the excessive use of antibiotics in public health and with productive activities such as aquaculture (Alatore et al. 2016). Environmental transfer of ARG can stimulate the presence of new multiresistant pathogenic strains, with consequent risks to public health (Gao et al. 2012). Previous reports have demonstrated that using wastewater for irrigation increases the presence of ARB (Palacios et al. 2017) compared with soils irrigated without using wastewater.

When considering the dispersion of ARB and ARG in the environment because of human-related activities, the impact presented is related to Human Public Health and the ecosystem equilibrium, and using the One Health approach can provide a systematic view. Although it is not a new concept, the multidisciplinary approach of One Health suggests providing a framework for scientists, governments, and international policymakers to suggest collaborative solutions (Mumford et al. 2023). One Health promotes sustainable actions for the problems that are included and are closely related to the objectives sought under the 2030 Sustainable Development Goals.

Therefore, we aimed to evaluate the presence of ARB and ARG in mangrove sediments of Bahia Magdalena, Mexico, to establish a relationship between the ARG and the bacterial isolates, as well as between the conditions of the sampling site and the presence of ARB and ARG.

## Material and methods

### Selected area and sediment sampling

**Figure 1.**
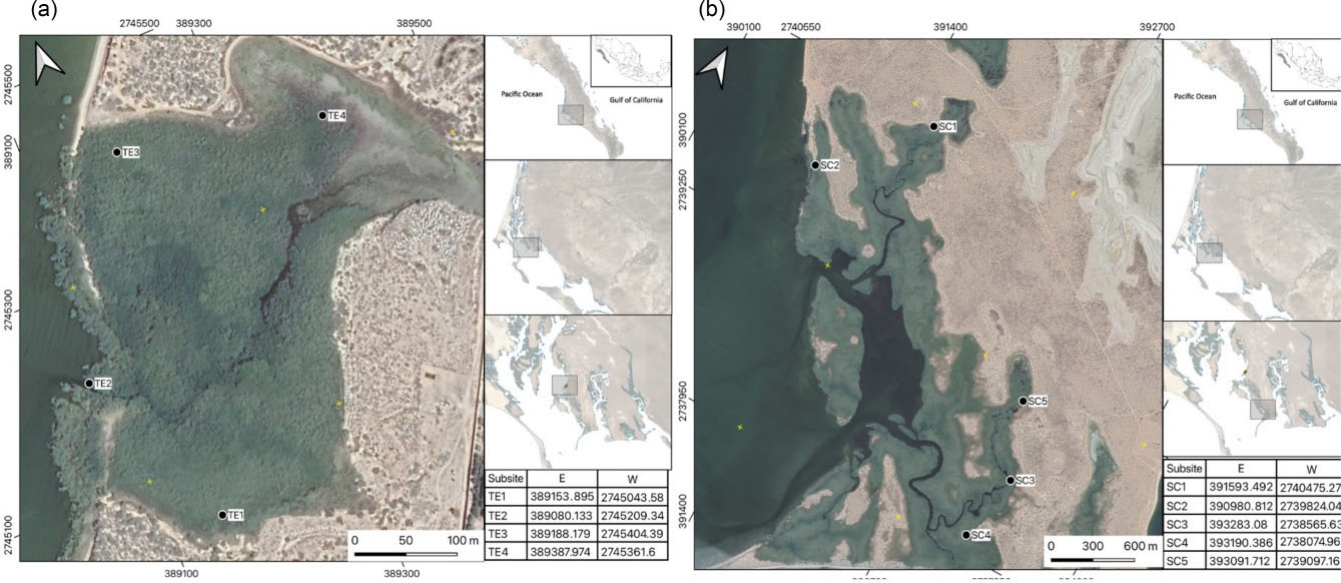
The Bahia-Magdalena-Almejas lagoon complex sampling sites. (a) Port San Carlos (b) thermoelectric power plant.

The study area was selected based on its proximity to an urban area with fishery, tourism, and commercial activities. The Bahia-Magdalena-Almejas lagoon complex is located in the Southeast part of the Baja California Sur Peninsula. The Bahía Magdalena mangrove communicates with the open sea and is also a navigation channel connecting to Puerto San Carlos. It is a protected natural designated area since it is an area of reproduction of the Gray Whale. Two sites were selected, as identified in Fig. 1. One of the sites, SC (Port San Carlos), is a zone of important fishery activity since it is close to Puerto Mateos and Puerto San Carlos. Therefore, the primary contamination is related to this activity. The site is not close to an urban setting. The other sampling point is near a thermal power station (TE), and the residues of this plant are released into the mangrove area.

Samples were taken with a sediment core sampler to three depths (0–15, 15–30, and 30–50 cm). Five subsites were selected in the SC area and four in the TE point; the location is included in Fig. 1. A subsample was preserved at −80°C for DNA extraction, and the rest of the sample was air-dried at room temperature and stored in plastic resealable bags (Palacios et al. 2017).

### Isolation of ARB from sediments

Decimal dilutions (1 × 10^−1^ to 1 × 10^−4^) of soil sediment samples were prepared by an initial 1 g of sediment suspension in 9 ml of sterile saline solution (0.154 M). Dilutions were inoculated by spread plate technique in TSA (Tryptic Soy Agar, BD Bioxon, Mexico city, Mexico) added with NaCl (0, 0.256, 0.513 M). Isolates were selected based on their colonial morphology difference and were obtained as pure cultures by re-streaking on TSA, with the same concentration of NaCl as in their initial isolation. The isolates were preserved in TSA slant tubes and stored at 4°C. The resistance to antibiotics was tested by inoculation of the strains in TSA plates containing the antibiotic at the following concentrations: Ampicillin (5.72 µM), cefotaxime (4.39 µM), ciprofloxacin (3.018 µM), vancomycin (1.38 µM), and tetracycline (4.5 µM). The antibiotics (Thermo Fisher Scientific, Waltham, Mass, USA) were prepared to an initial concentration of 10 000 µg/ml in either pure ethanol(100%) (cefotaxime, tetracycline) or distilled water (ampicillin, ciprofloxacin, vancomycin). The solutions were sterilized by filtration (0.22 µm pore membrane, Millipore). The concentrated solution was added to the appropriate volume to TSA supplemented with NaCl (0, 0.256, 0.513 M). The isolates were inoculated from a separated colony using a sterile toothpick, into the antibiotic-containing agar plates, with the NaCl concentration used in its initial isolation. The plates were incubated at 36°C for 24 h. After incubation, the isolates that were able to grow at the antibiotic concentration present in the TSA plates were considered resistant. Those isolates resistant to three or more antibiotics were considered multiresistant (Magiorakos et al. 2012).

### DNA extraction from bacterial isolates and mangrove sediments

Total DNA from multiresistant bacterial isolates was done using the ZymoBiomics Miniprep kit (Zymo Research, Irvine, CA. USA), following the manufacturer's instructions. For isolation of total DNA from sediments, the Fast DNA SPIN for soil kit was used (MP Biomedicals, Irvine, CA. USA), with few modifications, including a washing step with guanidine thiocyanate (4 M); also, the vortex time was increased to 20 min and the time of contact with the lysis buffer was increased to 5 min. DNA integrity was verified by electrophoresis in agarose gel (1%).

### Amplification of antibiotic resistance genes from total DNA bacterial isolates and mangrove sediments

Final point PCR was used to amplify the genes related to antibiotic resistance from total DNA obtained from bacterial isolates and mangrove sediments, based on Berendonk et al. (2015). The primers used have been previously used to evaluate the presence of ARG in environmental samples. The PCR assays were carried out in 20 μl reaction systems containing 1 μl dNTP (10 mM), 2.5 μl 5× PCR buffer, Taq polymerase (5 U/ml) (GoTaq Flexi DNA polymerase, PROMEGA, Madison, Wisconsin, USA), 1 μl of each primer (10 mM), and 20 ng template DNA. Primers (T4 Oligo, Irapuato, Gto. Mexico) and conditions used in this study are presented in Table 1. The thermal cycle procedure for each set of primers was as follows: For the *int*1 gene, initial denaturation of 95°C for 5 min, followed by 35 cycles of 94°C for 60 s, 67°C for 60 s, and 72°C from 60 s, with a final extension of 72°C for 5 min. For the *sul*1 and *sul*2genes, initial denaturation of 94°C for 5 min, followed by 30 cycles of 94°C for 60 s, 55°C for 60 s, and 72°C from 60 s, with a final extension of 72°C for 7 min. For the *bla*CTXM gene, conditions included an initial denaturation of 94°C for 5 min, followed by 35 cycles of 94°C for 60 s, 58°C for 120 s, and 72°C from 180 s, with a final extension of 72°C for 5 min. For the *bla*VIM and *bla*NDM genes, conditions included an initial denaturation of 94°C for 10 min, followed by 36 cycles of 94°C for 30 s, 52°C for 40 s, and 72°C from 50 s, with a final extension of 72°C for 5 min. For the *bla*KPC gene, initial denaturation of 94°C for 3 min, followed by 30 cycles of 94°C for 60 s, 52°C for 60 s, and 72°C from 60 s, with a final extension of 72°C for 10 min. For the *qnr*S gene, conditions included an initial denaturation of 95°C for 5 min, followed by 35 cycles of 95°C for 60 s, 54°C for 60 s, and 72°C from 60 s, with a final extension of 72°C for 5 min. For the *van*A gene, conditions included an initial denaturation of 96°C for 5 min, followed by 25 cycles of 55°C for 5 s, and 60°C for 60 s. Finally, conditions for the *tet*A gene were an initial denaturation of 94°C for 5 min, followed by 30 cycles of 94°C for 30 s, 55°C for 30 s, and 72°C from 30 s, with a final extension of 72°C for 5 min.

**Table 1.** Primers used in this study for PCR reactions to detect ARG in bacterial isolates and mangrove sediments.

Gene	Primer	Sequence(5′-3′)	Amplimer (bp)	Reference
*int1*	*int1*F	GTT CGG TCA AGG TTC TGG	923	(Cergole et al. 2011)
	*int1*R	GTA GAG ACG TCG GAA TGG		
*sul1*	*sul1*F	CGG CGT GGG CTA CCT GAA CG	433	(Hoa et al. 2008)
	*sul1*R	GCC GAT CGC GTG AAG TTC CG		
*sul2*	*sul2*F	GCG CTC AAG GCA GAT GGC ATT	293	(Hoa et al. 2008)
	*sul2*R	GCG TTT GAT ACC GGC ACC CGT		
*bla*CTXM	*bla*CTXMF	ATG TGC AGY ACC AGT AAR GT	593	(Shahid 2010)
	*bla*CTXMR	TGG GTR AAR TAR GTS ACC AGA		
*bla*VIM	*bla*VIMF	GTT TGG TCG CAT ATC GCA AC	621	(Poirel et al. 2011)
	*bla*VIMR	AAT GCG CAG CAC CAG GAT AG		
*bla*NDM	*bla*NDMF	GGT TTG GCG ATC TGG TTT TC	432	(Poirel et al. 2011)
	*bla*NDMR	CGG AAT GGC TCA TCA CGA TC		
*bla*KPC	*bla*KPCF	TGT CAC TGT ATC GCC GTC TAG	880	(Gootz et al. 2009)
	*bla*KPCR	TTA CTG CCC GTT GAC GCC CAA TCC		
*qnr*S	*qnr*SF	GCA AGT TCA TTG AAC AGG GT	428	(Matsumura et al. 2017)
	*qnr*SR	TCT AAA CCG TCG AGT TCG GCG		
*van*A	*van*AF	CTG TGA GGT CGG TTG TGC G	377	(Volkmann et al. 2004)
	*van*AR	TTT GGT CCA CCT CGC CA		
*tet*A	*tet*AF	AGT GGA GCG ATT ACA GAA	158	(Strommenger et al. 2003)
	*tet*AR	CAT ATG TCC TGG CGT GTC TA		

PCR products were analyzed by gel electrophoresis in agarose gels (1.2%) for 90 min at 90 V. As positive controls, clinical isolates that were previously reported as antibiotic-resistant were used, with the following phenotype: *Escherichia coli* 37 Hz (*bla*VIM +, *bla*kNDM +), *E. coli* 22 Hc (*int1 +, sul1 +, sul2 +, bla*CTX-M +, *bla*KPC +), *E. coli* 61 HC (*qnr*S +), *E. coli* 2 HZ (*van*A +) and CFE3 0–15 10^3^ 3 (*tet*A +). To visualize the amplified DNA, gels were stained with ethidium bromide (0.5 µg/ml).

### Phenotypic profile of multiresistant bacterial isolates

A cluster analysis was done to select only those isolates that were different based on their phenotypic profile for antibiotic resistance, discarding duplicated bacterial isolates. The analysis considered the sampling subsite and the coded resistant profile (1 for resistant, 0 for susceptible). The conditions used in the cluster analysis were a complete hierarchical analysis using Euclidean distances and a 95% similarity percentage. Pearson correlation coefficients were also calculated from the codded data to assess the relationship between antibiotic resistance and ARG in the bacterial isolates. The statistical analysis was done using Minitab ver 18 statistical software (Minitab, Inc., State College, PA, USA).

## Results

### Sampling Site and anthropogenic activities

Samples were taken in November 2019 in Bahia Magdalena, Baja California Sur, as described in Fig. 1. A total of 30 samples were used in the study to obtain DNA from the sediment and to isolate bacterial strains that were tested for antibiotic resistance, as described in the methodology section. San Carlos Port's economic activities include fishery and tourism since it is known to be an area of reproduction of the Gray Whale (Fig. 1A). The second site was the mangrove close to the thermoelectric generation plant, where the mangrove was physically affected by the effluents of the industrial facility (high temperature of the effluent).

### ARG in mangrove sediments

The presence of ARG in sediments was carried out for the integron1 gene and the genes included in Table 1. Five samples were analyzed for each site and depth; Table 2 presents the number of samples with a positive result for the presence of each of the genes analyzed. Therefore, the higher the number, the higher the gene abundance in the mangrove sediments. The data could not be analyzed for significant differences since they do not meet the criteria for Chi-square analysis (a high number of observed frequencies below 5). Still, it can be observed that there was no difference in the presence of ARG between the two sites included. However, the depth at which the sediment was taken can affect the presence of the different ARG genes. The *int*1 gene that codifies for the integron Class I was not present in lower sediments, while other genes were present in more samples at 15–30 cm deep or even in 30–50 cm deep sediments (*bla*CTX-M, *sul*1, *sul*2).

**Table 2.** Detection of ARG in sediment samples at three different depths in two locations of Bahia Magdalena, Mexico mangrove.

	Site and sampling depth (cm)					
		SC			TE	
Genes	0–15	15–30	30–50	0–15	15–30	30–50
int 1	5	4	0	5	5	0
*sul* 1	3	3	1	5	4	2
*Sul* 2	5	4	1	5	5	4
*bla*CTX-M	2	3	2	1	4	2
*bla*KPC	2	0	0	3	3	0
qnrS	2	3	0	3	3	1
*Tet*A	2	3	1	3	3	1
*bla*VIM	0	2	1	0	3	1
*bla*NDM	0	3	1	1	3	1
*van*A	4	3	1	5	3	1

**Figure 2.**
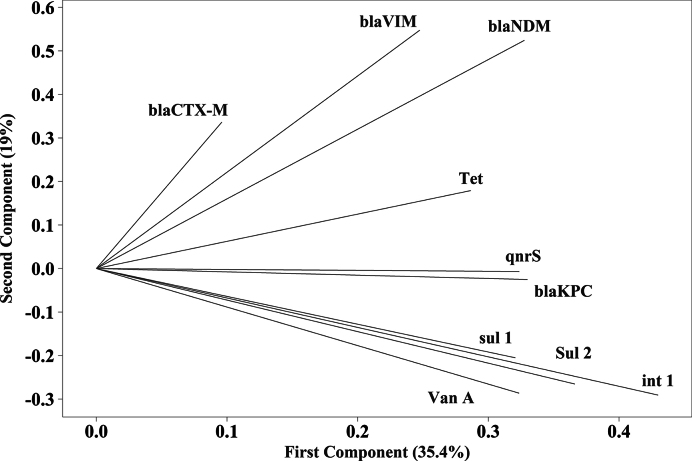
Principal components analysis (PCA) loadings of the first two components on the presence of ARG in soil sediments from Bahia Magdalena, Mexico mangrove.

A PCA (principal component analysis) on the presence of ARG genes was done to identify the grouping of ARG present in the mangrove sediments. A 54.4% of the variability was accounted for in the first two components, with 35.4% for PC1 and 19% for PC2. All the genes had a positive load for PC1, while *sul*1, *sul*2, *van*A, and *int*1 had a negative load in PC2; load for *bla*KPC and *qnr*S for PC2 was close to 0, and was positive for the rest of the ARG. Therefore, the presence of the integron gene and those related to sulfonamides and vancomycin were closely related (Fig. 2).

### Phenotypic and genotypic profile of antibiotic-resistant isolates from mangrove sediments

**Figure 3.**
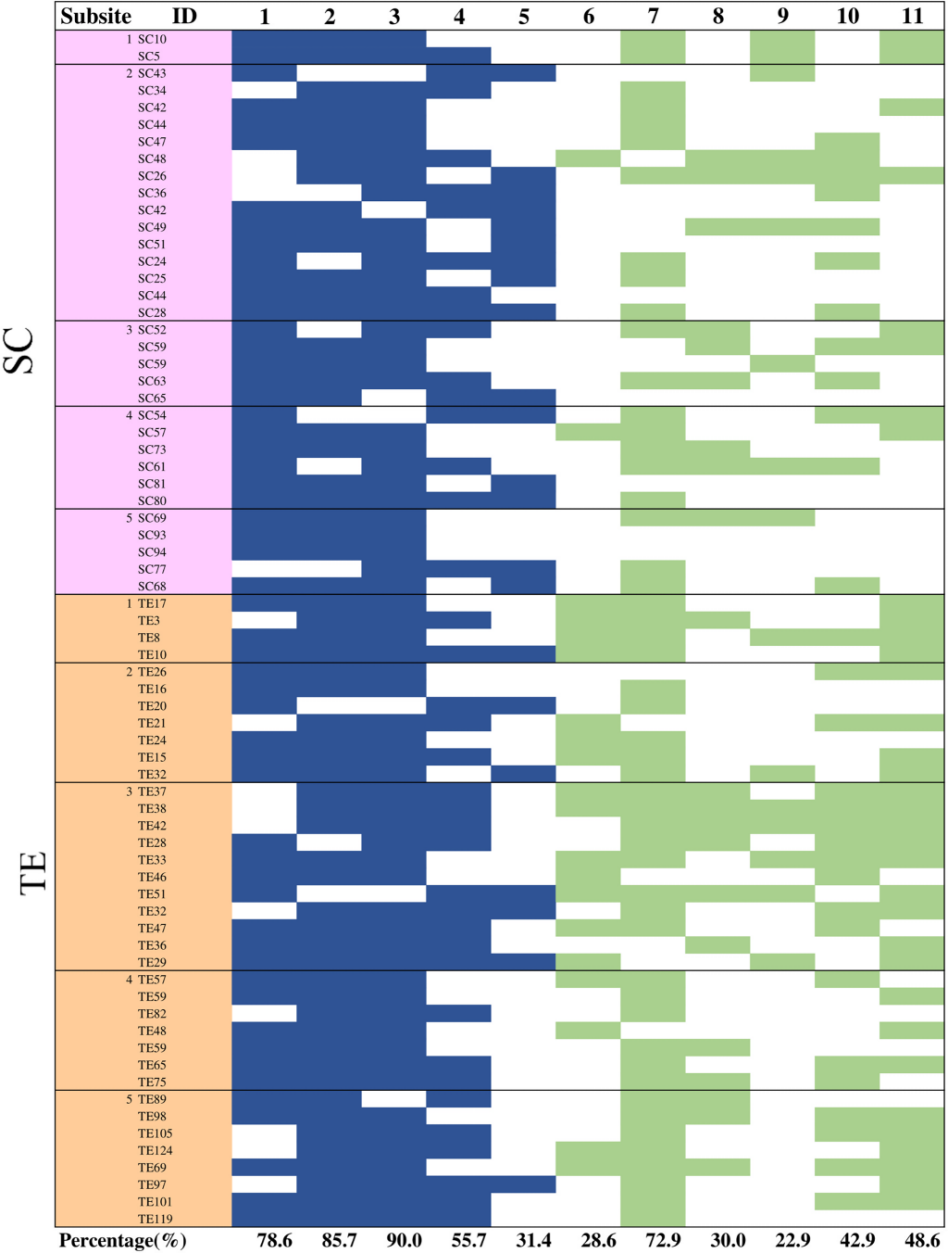
Phenotypic and genotypic profile of bacterial isolates from soil sediments from Bahia Magdalena, Mexico mangrove. Antibiotic resistance. (1) Ampicillin (5.72 µM), (2) Cefotaxime (4.39 µM), (3) Ciprofloxacin (3.018 µM), (4) Vancomycin (1.38 µM), (5) Tetracycline (4.5 µM). Presence of genes in total DNA (6) *int*1, (7) *bla*CTX-M, (8) *qnr*S, (9) *tet* A, (10) *bla*NDM, and (11) *van*A.

From the soil sediments, a total of 70 bacterial isolates were considered multiresistant since they were resistant to three or more of the following antibiotics: ampicillin, cefotaxime, ciprofloxacin, vancomycin, or tetracycline at the concentrations tested. The antibiotics were selected based on their mechanism of action and the prevalence of their use in human health or food-producing industries. The presence of ARGs related to those antibiotics was also tested on the bacterial isolates, including the one for the integron Class 1 (*int*1) the *bla*CTX-M, *qnr*S, *tet* A, *bla*NDM, and *van*A genes. The phenotypic and genotypic profile of the bacterial isolates is shown in Fig. 3, where antibiotic resistance is marked in blue, and the presence of ARG is marked in green. A similar number of isolates were tested for the SC (33) and TE (37) sites. The strains were more resistant to ciprofloxacin (90%), cefotaxime (85.7%), and ampicillin (78.6%), while the beta-lactamase gene (*bla*CTX-M) was present in 72.9% of the tested isolates. Only in 28.6% was the integron Class 1 present. The phenotypic and genotypic profile of the bacteria isolates from the sediments showed no clear relation between antibiotic resistance genes and antibiotic resistance behavior (Fig. 3).

The relationship between the genotypic and phenotypic profile of antibiotic resistance was evaluated by determining the Pearson correlation coefficient, as presented in Table 3. The antibiotic resistance profile of the isolates showed a positive correlation between cefotaxime resistance and the resistance to ciprofloxacin (*P* < 0.01), while a negative correlation was significant between cefotaxime and vancomycin and tetracycline (*P* < 0.01). Moreover, no correlation was significant between antibiotic resistance genes and the antibiotic resistance behavior of bacterial isolates, regardless of the sampling point (CS or TE). A positive correlation was found between the presence of the Integron Class I gene and the *van*A gene, for vancomycin resistance (*P* < 0.01).

**Table 3.** Relationship between antibiotic resistance phenotype and antibiotic resistance genes in bacterial isolates from sediments of Bahía Magdalena mangrove, Mexico.

	Ampicillin	Cefotaxime	Ciprofloxacin	Vancomycin	Tetracycline	*int* 1	*bla*CTX-M	*qnr*S	*tet* A	*bla*NDM
Cefotaxime	−0.01									
Ciprofloxacin	−0.17	0.41**								
Vancomycin	−0.40**	−0.36**	−0.30*							
Tetracycline	−0.02	−0.34**	−0.40**	0.17						
*int*1	−0.13	0.17	0.10	−0.01	−0.22					
*bla*CTX-M	−0.08	−0.06	0.12	0.10	−0.14	0.03				
*qnr*S	−0.11	−0.09	0.01	0.08	−0.24*	0.00	0.12			
*tet*A	−0.05	−0.07	−0.05	−0.06	0.07	0.11	−0.05	0.24*		
*bla*NDM	−0.18	−0.06	0.19	0.08	−0.09	0.09	0.07	0.25*	0.08	
*van*A	−0.19	0.07	0.13	0.06	−0.17	0.33**	0.21*	0.11	0.15	0.14

Pearson correlation coefficients. Asterisks indicate a statistically significant correlation.

* *P* < 0.05 ** *P* < 0.01.

## Discussion

Among the most pressing problems related to human health is the appearance of multidrug-resistant pathogens that can lead to a devastating preantibiotic era of infectious diseases. The appearance of ARB started just a few years after the widespread use of penicillin and continues to present strains with resistance to even the most recently used antibiotics. The different mechanisms of antibiotic resistance can be explained by the continuous adaptation of microorganisms to stressful environmental conditions, which can be considered as microevolution events. The genetic information of antibiotic resistance is carried out in plasmids, which can be easily transferred along bacterial cells not only of the same family but even of different families. Therefore, dissemination of the genetic information of antibiotic resistance can easily occur. Pathogenic bacteria carrying ARG can be released into the ecosystem and transfer their genetic information to environmental bacteria; then environmental bacteria are now ARG reservoirs (Andersson et al. 2020).

Although many environments have been analyzed for ARG and ARB, mangroves are one of the less-studied ecosystems (Palacios et al. 2021). The One Health approach to solving the antibiotic resistance challenge highlights the contribution of antibiotics used in farming and aquaculture, being as important or more important than the abuse of antibiotics in human health care (Larsson and Flash 2022). Most of the reports on the dispersion of ARG and ARB in mangroves are from Asia, with almost no reports from America. Moreover, the mangrove ecosystem from Baja California and Sonora, Mexico, is particularly interesting since they are in a region where temperatures hardly allow for mangrove growth (López-López 2013, Velázquez et al. 2021). The rural and urban settings close to the mangroves and the economic activities developed in the area can impact the release of antibiotic residues, ARB, and ARG.

We selected two sites in the Bahia Magdalena area on the Pacific Coast of Baja California Sur, Mexico: one that was closer to an urban setting (Port San Carlos) and another closer to a thermoelectrical plant that showed signs of mangrove physical deterioration (TE). Many factors can be associated with the increment in antibiotic resistance in environmental bacteria, including the persistence of antibiotic residues, the composition of the microbial community in the site, or the accumulated effect of anthropogenic activities (Bottery et al. 2021). Although anthropogenic activities could significantly impact the TE point, results showed no difference in the presence of ARB and ARG between the two sites.

The distribution of microbial species in a particular microenvironment is linked to their ability to endure the environmental stress in that habitat. In shallow mangrove sediments, oxygen concentration and nutrients allow for the survival of more bacterial species, primarily aerobic or facultative. It is anticipated that as the depth of the sediments increases, the diversity of microorganisms will decrease. A similar trend is seen in the distribution of ARB and ARG. With the sampling procedure we use, samples at different depths could be obtained; therefore, the effect of depth on the presence of ARB and ARG was also tested. The effect of depth on ARG has been reported to be negligent since ARB harboring the ARG can efficiently propagate these genes through the vertical sediment layers (Ohore et al. 2020). Nevertheless, in some environments, deeper sediments will have a lower oxygen pressure, which induces a harsh environment for the survival of ARB, with the consequent decrement in ARG prevalence (Ohore et al. 2019). Contrary to our findings, Tan and coworkers (2019) reported an increment of relative abundance of ARG from the top to the deep layer. While working with arid soils, these authors relate their findings to the decrease in salinity observed in the deepest sediments and the decreased presence of actinobacteria. We consider that the number of ARB can be higher in the shallow sediments, where aerobic bacteria are predominant.

Regarding the presence of the ARG in mangrove sediments, it was observed that the Class 1 integron was commonly found in the two mangrove ecosystems analyzed. This integron class has been related to clinical isolates of Gram-negative antibiotic-resistant strains and is responsible for acquiring and disseminating ARG (Zhu et al. 2017). In marine sediment, this integron class has been commonly related to sulfonamide resistance genes (Chen et al. 2015), but genes related to vancomycin resistance have also been reported to be clustered with class 1 integron (Yang et al. 2019). Additionally, it is well known that vancomycin resistance genes are ancient in the environment (Zhu et al. 2017), explaining their higher prevalence in both mangroves.

From the sediments, ARB isolates were tested for their phenotypic antibiotic profile; at the same time, the presence of the ARG was assessed by PCR. The results showed that the resistance to antibiotics tested was not correlated to the presence of ARGs. Various mechanisms, such as ion channels and efflux pumps, may be responsible for the phenotypic profile of bacterial isolates. Efflux pumps have been reported as the predominant mechanism for antibiotic resistance in bacteria from marine sediment. This is because efflux pumps can also help extrude other compounds that could adversely affect bacterial growth, thus acting as a detoxifying mechanism (Xiao et al. 2016). On the other hand, Tan and coworkers (2019) reported a decrement in ARG and MGEs when high salinity (3.5% NaCl) was present. The authors propose that the increment of saline stress can avoid the dissemination and proliferation of ARG in the marine environment since salinity can promote the elimination of antibiotic-resistant plasmids (Tan et al. 2019).

Subinhibitory antibiotic concentrations in the environment can drive the evolution of antibiotic resistance in environmental microorganisms. The concentration of antibiotics in soil and water ecosystems varies depending on the contaminants discarded in those environments. As such, the minimal inhibitory concentration (MIC) of bacterial isolates from aquatic environments can vary from 10 (sea and rivers) up to 10 000 µg/l (industrial polluted surface water) (Larsson and Flach 2022). Bengtsson-Palme and Larsson (2016) proposed the Predicted No Effect Concentrations (PNECs) as the concentration of antibiotics that does not impact the generation of antibiotic-resistant microbial cells. PNECs were predicted from the MIC values of environmental isolates for more than 100 antibiotics and were in the range of 8 ng/L–64 μg/L (Bengtsson-Palme and Larsson 2016). The concentration of antibiotics used for identifying resistant bacteria in this study is up to 100 times higher than the proposed PNECs. However, they are lower than those used for clinical classification purposes.

Further studies on different environments regarding antibiotic resistance dissemination and the strategies used by bacteria for survival can provide strategies to cope with the problem in Human Health. The multidisciplinary One Health approach needs input from as many disciplines as possible to integrate a global view of the problem. In the end, if no actions are taken, the information will not help achieve the goal of restoration of the environmental equilibrium (Mumford et al. 2023).

Many questions still need to be answered in the dispersion of ARB and ARG in the mangrove ecosystem. Since the mangrove can function as a purifying environment, it is suggested that the ARGs are diminished in the ecosystem (Palacios et al. 2021). The quantification of antibiotic residues in the mangrove sediments can also provide clues on the behavior of antibiotics and their impact on the resistome as a possible source of ARB dissemination.

## Conclusions

Samples of mangrove sediments collected from Bahia Magdalena in Baja California Sur, Mexico, showed the presence of ARB and ARG in two areas located at varying distances from human settlements. Notably, the prevalence of ARG was observed even at depths of 30–50 cm. Based on PCA analysis, the *int*1 gene of Class I integron was correlated with genes related to sulfonamides and vancomycin resistance. Salt concentrations ranging from 0–0.513 M in TSA were used to select 90 multiresistant bacterial isolates based on their phenotypic profile during the initial isolation procedure; the presence of ARG was also tested. However, the Pearson correlation did not indicate a significant correlation between the presence of ARG and the antibiotic-resistant profile of the bacterial isolates. This suggests that other antibiotic-resistance mechanisms may be at play. Interestingly, despite differences in anthropogenic activities between the two sites studied in the Bahía Magdalena region, there were no differences in the presence of ARB and ARG.

## Data Availability

The data underlying this article will be shared on reasonable request to the corresponding author.
